# How Long Do Octogenarians Benefit From Knee Arthroplasty?

**DOI:** 10.7759/cureus.14997

**Published:** 2021-05-13

**Authors:** Can Doruk Basa, Elcil Kaya Bicer, Semih Aydogdu, Hakki Sur

**Affiliations:** 1 Department of Orthopaedics and Traumatology, Tepecik Training and Research Hospital, Izmir, TUR; 2 Department of Orthopaedics and Traumatology, Ege University Faculty of Medicine, Izmir, TUR

**Keywords:** octogenarians, total knee arthroplasty, mortality survey, geriatry, knee

## Abstract

Introduction

Elderly patients are more prone to surgical risk regardless of the procedure. The overall mortality rate is expected to be high in this population. The aim of this study was to evaluate the survival rates of octogenarians who underwent knee arthroplasty procedures.

Methods

Sixty-two knee arthroplasties were performed on 52 patients who were >80 years of age at the time of the operation between November 1996 and May 2014. The preoperative American Society of Anesthesiologists (ASA) classes were available for 45 procedures. The database of the Civil Registry Service was used to assess whether the patients were alive at the time of the study. If they were deceased, their dates of death were recorded. The five-, 10-, and 15-year survival rates of patients were determined.

Results

Thirty patients (57.69%) were alive and 22 (42.31%) were deceased at the time of analysis. Based on the 62 procedures, the mean age of the patients at the time of the operation was 82.56 ± 2.18 years. The mean time span between the operation and death of patients who passed away was 6.4 ± 4.66 years. The mean age of the patients who were alive at the time of the study was 86.63 ± 3.60 years. The mean time that had passed since the operation was 4.41 ± 2.9 years for living patients. Only one patient died during the first 90 days postoperatively. The one-year mortality rate was 4.84% (three patients). A Kaplan-Meier survival analysis revealed that the mean survival time of the patients was 6.4 years, and the median survival time was 5.6 years. The five-year survival rate was 59%, the 10-year rate was 19%, and the 15-year rate was 7%.

Conclusion

Octogenarians benefitted from knee replacement longer than expected. Early mortality risks can be avoided with proper patient selection.

## Introduction

Total knee arthroplasty (TKA) is a common and satisfactory treatment option for end-stage knee osteoarthritis [1]. Both TKA and total hip arthroplasty rates have been increasing over the last 20 years [2-3]. The number of TKA surgeries is gradually increasing in both young and elderly populations [4]. However, there are concerns in both age groups, including fear of early failure, particularly in younger patients. Although some studies have shown that arthroplasty in younger populations is as beneficial as in older populations [5-6], the common view is that revision rates are high [7-8]. However, elderly patients have early and late complications.

As longevity increases, elderly patients require a more painless and higher quality of life [9]. Around 80% of patients undergoing total knee replacement are satisfied with their operations [10]. Surgeons prefer easier surgical treatments [11-12]. Complications are manageable due to improved medications and medical technology, which have considerable influence on the choice of surgical treatment. However, elderly patients are more prone to surgical risk regardless of the procedure [9,13]. Early mortality is a feared complication, particularly in elderly patients. One study showed that all systemic and local minor or major complications, including death in the first 90 days, increased in patients over 85 years in age [14]. Thus, surgeons usually choose nonoperative treatments for octogenarians with end-stage knee osteoarthritis.

Another problem in elderly patients is that we are unaware of how long the patient will use the prosthesis, as we do not know when the patient will die. In general, surgeons commonly believe that patients >80 years of age will die within several years, thus the operation is unnecessary from a medical and economic point of view. Most surgeons refrain from operative treatments in this age group, as they are hesitant about early complications and life expectancy is presumed to be short. However, as a patient’s pain does not decrease and the quality of life does not change with non-surgical treatment, this option leaves patients to suffer through their pain.

The aim of this study was to evaluate the survival rates of octogenarians who underwent the knee arthroplasty procedure.

This article was previously presented as a meeting abstract at the German Congress of Orthopaedics and Traumatology (DKOU 2015) on October 23, 2015 (https://www.egms.de/static/en/meetings/dkou2015/15dkou015.shtml).

## Materials and methods

All patients who underwent TKA in our clinic between November 1996 and May 2014 were identified retrospectively. A total of 1,415 patients and 1,476 knees (61 bilateral) were operated on for total knee replacement in university hospitals' orthopedics and traumatology clinic. Of the patients, 1,232 were female and 183 were male. Patients ≥80 years of age who had undergone either revision or primary TKA were included in this study. Patients who did not have sufficient clinical and identity data were excluded from this study. Sixty-two knees (52 patients) were ≥80 years of age at the time of surgery. The mean age of the patients was 82.56 ± 2.18 years (80-89 years) at the time of the operation. Seven patients were male and 45 were female. Of these 62 procedures, five were revisions and the remainder were primary TKAs. All these five revision procedures were for aseptic loosening. Ten patients had bilateral knee arthroplasty; only one of these procedures was a simultaneous operation. All prostheses were cemented, and the posterior cruciate ligament was retained. None of the primary prostheses in this study were revised.

Preoperative American Society of Anesthesiologists (ASA) grades were obtained from the anesthetic chart; 45 patients had an available ASA grade. The Civil Registry Service database was accessed to determine whether patients were alive at the time of the study. If a patient was deceased, their date of death was recorded. The mortalities of the patients in the first 90 days and the first year were evaluated. Mortalities in the first 90 days were regarded as the early period and those after the first year as late period mortality. The correlation between ASA grade and the patient survey was evaluated. The five-, 10-, and 15-year survival rates were determined.

The protocol for collecting the data of human subjects was approved (Institutional Review Board) and the study was conducted in accordance with the standards of the Declaration of Helsinki (Finland).

Statistical analyses were performed utilizing the Statistical Package for the Social Sciences (SPSS) v. 18 software (SPSS Inc. Released 2009. PASW Statistics for Windows, Version 18.0. Chicago). Descriptive statistics, t-tests, correlation analysis, Kaplan-Meier survival analysis, and survival analysis using life tables were conducted. A p-value < 0.05 was considered significant.

## Results

Eight patients had an ASA grade of 1, 36 had a grade of 2, and one patient had a grade of 3. At the time of the study, 30 patients (57.69%) were alive and 22 (42.31%) were dead. Four out of 10 of the bilateral cases were dead. Based on 62 procedures, the mean age of the patients at the time of the operation was 82.56 ± 2.18 years (range, 80-89 years). The mean age of the patients at the time of death was 88.63 ± 3.95 years (range, 82-99 years). The mean time span between the operation and death was 6.4 ± 4.66 years (range, 30 days to 17.50 years). The mean age of the patients alive at the time of the study was 86.63 ± 3.60 years (range, 82-96 years). The mean time that had passed since the operation (based on 35 procedures) was 4.41 ± 2.9 years (range, 3 months to 13.10 years) for the patients who were still alive (Table 1).

**Table 1:** Summary of the study data

Health status at the time of study n (%)	
Alive	30 (57.7%)
Dead	22 (42.3%)
Age at the time of operation	82.6±2.2 years (range, 80–89 years)
Age at the time of death	88.6±4 years (range, 82–99 years)
Timespan between the operation and death	6.4±4.7 years (range, 30 days to 17.50 years)
Age of the patients alive at the time of study	86.6±3.6 years (range, 82–96 years)
The mean time that had passed since the operation	4.4±2.9 years (range, 3 months to 13.1 years)

**Figure 1:**
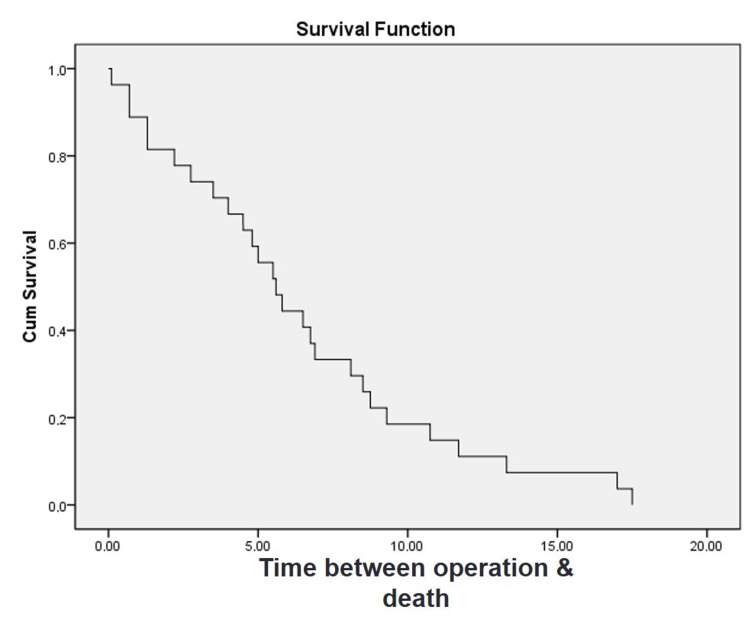
Kaplan–Meier survival curve with death as the endpoint

The mean age of the living patients at the time of operation was not significantly different from that of the deceased patients (t-test, p = 0.749). Only one patient died during the first 90 days postoperatively (1.61%). The one-year mortality rate was 4.84% (three patients). Three patients with first-year mortality did not have minor or major orthopedic complications. There was no association between the postoperative complications of TKA and early mortality. The Kaplan-Meier survival analysis revealed that the mean survival time of the patients was 6.4 years (95% confidence interval [CI], 4.644-8.156) and the median survival time was 5.6 years (95% CI, 4.243-6.957) (Figure 1). The five-year survival rate was 59%, the 10-year rate was 19%, and the 15-year rate was 7%. The correlation between the ASA grades of 45 patients and their survival was evaluated. A moderate negative correlation was observed between the ASA grade and survival (r = −0.344; p = 0.021). 

## Discussion

Before we conducted this study, we wondered how long octogenarians used their prostheses after the operation. The mean duration of prosthetic use in this study was 6.4 years in patients who died before the study, and 4.4 years in patients who were alive during the study. Although many studies have been performed regarding mortality and complications, no study has emphasized how long patients used their prostheses.

Miric et al. analyzed whether a knee prosthesis could be safely implemented in patients >90 years of age. In that study, mortality in the first 30 days (1.4%) and the length of hospitalization were highest in the group >90 years of age (p< 0.001) [15]. The authors found an annual mortality rate of 5.6% and observed that the annual mortality of nonagenarians was lower than that reported in other studies.

In the present study, the correlation between the preoperative ASA grade and the survey was evaluated. A significant negative correlation was detected between the ASA grade and the survey. Higuera et al. evaluated the risk factors for early side effects in patients who underwent knee and hip arthroplasty [14]. In that study, early mortality occurred in five (1%) patients out of 502 during the first 90 days. In our study, only one mortality was observed during the first 90 days, which was similar to the results reported by Higuera et al. In their study, Higuera et al. used the Charlson Comorbidity Index (CCI) rather than ASA grade to assess comorbidities. They showed that length of hospitalization and all systemic complications were correlated using the CCI. Although complications and duration of hospital stay were not evaluated in the present study, ASA grades were used to assess comorbidities, and a negative correlation was found with the patient survival.

The ASA grade was also used in a study by Parvizi et al. [16] to assess early mortality. ASA grade was significantly associated with early mortality (P < 0.0001). This result is similar to our findings. Patients with higher ASA grades are evaluated as higher risk in terms of early mortality [16].

Parvizi et al. [16] evaluated patient mortalities in the first 30 days after receiving a total knee replacement. A total of 47 (0.21%) mortality cases were observed out of 22,540 patients. Three of these patients died during the operation and the others died during the postoperative period. Of the patients who died, 39 were >70 years of age. Being >70 years of age was a significant risk factor in terms of early mortality (P < 0.005). No mortality was observed in patients who were fitted with a cement-free prosthesis. They observed that the mortality rate of patients who were treated bilaterally was significantly higher than that of patients who were treated unilaterally. In the present study, the first 90 days were used to evaluate early mortality. Although we performed our study in an age group that was much higher than that assessed by Parvizi et al., no mortality was observed during the surgeries. Cemented total knee prostheses were used in all patients. Therefore, we cannot comment on the correlation between the use of cement and early mortality in this study. In our study, the annual mortality rate was 4.84%, or three patients, but we observed only one mortality during the first 90 days. Considering that the patients in the present study were >80 years of age, the presence of only one mortality case in the first 90 days, and the fact that the annual mortality rate was 4.84% with three patients, and none of these patients were treated bilaterally, indicates that early mortality rates are not as high as predicted.

The lack of a control group and the small number of patients were the limitations of the study. Also, it was based on analyses of civil registry data, which is why clinical results, functional assessments, and quality of life analyses were beyond the scope of this study. Assessment of these parameters and of the cost-effectiveness of total knee arthroplasty in octogenarians should be the subjects of future studies.

## Conclusions

TKA in octogenarians is applied with hesitation due to the low life expectancy of the patient. This group of patients can use prostheses for an average of 6.4 years after the operation. Contrary to what was expected, early mortality was low and patients continued their lives with prostheses in this life period. Early mortality rates for TKA in octogenarians are not as high as to be expected. It should not be forgotten that patients use their prostheses for a considerable amount of time.
